# Forgetting tracked by recognition of pictures

**DOI:** 10.1177/17470218211047862

**Published:** 2021-10-04

**Authors:** Donald Laming

**Affiliations:** Department of Psychology, University of Cambridge, Cambridge, UK

**Keywords:** Forgetting, interference, Jost’s law, recognition memory, unequal variance/dual-process controversy

## Abstract

Thirty-three participants viewed 1,000 pictures for 6 s each. Recognition was tested after 10 different intervals of time by mixing 100 of the original 1,000 with 100 new pictures. Participants judged each test picture “Old” or “New” on a 6-point scale. The unequal-variance recognition model is reinterpreted to estimate the probability of retrieval of an original (1,000) picture after each lapse of time. A second model then relates those different estimates of accessibility to the lapse of time, taking into account the interference on each test from pictures presented in preceding tests. Studies of category judgement explain (a) why the model distributions are normal, (b) why the operating characteristics are asymmetric, (c) why they are curvilinear, and (d) why the asymmetry decreases with lapse of time, this to justify a particular estimate of accessibility (probability of retrieval). Nine candidate functions are shown to the accessibilities. The underlying relation is a power law, but the exponent is poorly determined by the data (−1.5, −0.5), as also is the offset from the temporal origin. Comparisons with previous work identify two different relationships with respect to lapse of time: The retrieval of a unique image shows an approximately reciprocal loss, whereas a decrease in the amount of material reproduced by recall, recognition, or other method is approximately logarithmic. The present experiment exhibits both relationships, depending on whether specific account is taken of the effects of interference or, alternatively, interference is entirely ignored.

## Introduction

One of the first questions addressed in memory research concerned the rate of forgetting (Ebbinghaus, 1885/1964). It is generally found that the probability of recall decreases with lapse of time; reminiscence (Ballard, 1913; Roediger & Thorpe, 1978; for reviews, see Erdelyi, 2010; Payne, 1987) is possibly an exception. But what is the functional form of that relation?

The experiment reported here sought to answer that question over the medium to long term in the hope that more precise knowledge of that functional form would be informative with respect to the mechanism of forgetting. The experiment was designed in 1983 with the function *f*(*t*) = *a* − *b.*ln(*t*) in mind (cf. Finkenbinder, 1913; Strong, 1913; Ebbinghaus, 1885/1964; Luh, 1922; see also Figure 7). But physical processes do not unfold in logarithmic time and, for this particular reason, a more accurate determination of the course of forgetting might have provided a valuable insight. Rubin and Wenzel (1996) have surveyed a century of work on this issue. They examined 210 published sets of data and fitted 105 different two-parameter functions to each set. They reported “The best fits were to the logarithmic function [*f*(*t*) = *a*−*b.*ln(*t*)], the power function [*f*(*t*) = *bt*^−α^], the exponential in the square root of time [*f*(*t*) = *a*exp(−α√*t*)], and the hyperbola in the square root of time [*f*(*t*) = 1/(*a* + *b*√*t*)]. It is difficult to distinguish among these 4 functions with the available data, . . . the same set of 4 functions fit most data sets” (p. 734).

This experiment was conceived in the hope of discovering the functional form of the relation between lapse of time and failure of recognition. But, anticipating the results, that was overambitious. Instead, a set of similar functions are compared to reveal, first, the general trend of the relation and, second, how precisely that trend is determined by the data. The functions compared are elementary, ideally with no free parameters (1/√*t*, 1/*t*; though a parameter is always introduced through normalisation) and, of course, the functions identified by Rubin and Wenzel. But, anticipating the results a second time, most of the functions compared do not have a finite integral with respect to time over the range from 0 to infinity. We are accustomed, subjectively, to recalling *a* memory and this feeds into the way countless experiments are analysed. But a function integrating to infinity with respect to time cannot be scaled to a probability distribution. Some of Rubin and Wenzel’s functions [*f*(*t*) = *a* − *b.*ln(*t*), *f*(*t*) = *bt*^−α^ for α ⩽ 1, *f*(*t*) = 1/(*a* + *b*√*t*)] fail to satisfy this requirement. To show that a representation in probabilistic terms is nevertheless feasible, the function *f*(*t*) = (α/√2π).*t*^−3/2^exp{−½α^2^*/t*} is also included. This is the strictly positive stable density of order ½ (Feller, 1966, p. 51), which has a number of applications in probability theory as the limit distribution for recurrence times and random walks.

There are four problems in tracking the rate of forgetting, especially when reanalysing historic data:

1. What dependent variable should be examined in relation to the lapse of time? Different procedures may be used to test retention—recall, recognition, reconstruction (of a list of words), savings (on relearning), and others (see Rubin & Wenzel, 1996)—and the different measures of retention may not be parallel with respect to time elapsed. For example, the number correct on a first relearning trial and the number of trials to relearn give different retention scores (Krueger, 1929).
2. If stimulus material is learned to criterion, there will be multiple sources in memory from which retrieval might be effected. It cannot ordinarily be known which of those sources is operative in any particular recall (cf. Laming, 2006, 2008) and the loss function will be different to that from a single source. If the *retrievals* from different sources are independent—not the memories, but the retrievals—the probability of recall is [1 − Π_*i*_(1 − *a_i_*)], where *a*_1_, *a*_2_, *a*_3_ and so on, are the individual accessibilities.
3. Multiple sources also introduce uncertainty about the actual time since presentation, though this ceases to matter when retention is tested after a long delay.
4. An accurate test of memory requires a sufficient volume of stimulus material with the possibility of confusion between different elements. In addition, the use of words poses a particular risk of artefact, because, of necessity, participants have much previous experience with words (Osth & Dennis, 2015).

In the light of these problems, I chose to use pictures as stimulus material, to test memory by recognition, and to model *accessibility*, that is, the probability of retrieving a specific image from memory. It is easy to assemble a collection of photographs that the participants have certainly not seen before, and individual pictures are readily recognised on test (Brady et al., 2008; Konkle et al., 2010; Nickerson, 1965; Standing, 1973). If a picture presented on test is to be recognised as having been seen before, it is necessary for the participant to retrieve a matching image, a sufficiently good match, from memory. Accessibility is the probability of retrieving that image and is measured by

(1) Accessibility=P(′old′|old)−P(′old′|New)

Suppose there is no retrieval, so that there is no basis for distinguishing Old from New. Then P(“Old”|Old)–P(“Old”|New) = 0, and this relation holds irrespective of the relative frequencies of Old and New pictures. If now a proportion *a* of Old pictures are recognised, then P(“Old”|Old) is increased by that amount and Equation 1 follows. Whatever procedure is used to measure retention, memories dating from some particular time previous have to be retrieved, so that accessibility is relevant for all procedures. With pictures the test can be focused on a single image in memory presented at a particular point in time. This finesses the problem of multiple images in memory. Nevertheless, there remains a problem of interference.

The experiment used 1,000 colour slides as stimuli. For practical reasons, the same participants were each tested after 10 different lapses of time, extending up to 4 months (cp. Nickerson, 1968; Shepard, 1967 [see Figure 6], and Fisher & Radvansky, 2018, who all tested different sub-groups of participants after each delay), and each test involved the presentation of further pictures that interfered with recognition on subsequent tests. This problem is addressed by modelling the interference as well as the recall of the original presentation, using the same loss function *f*(*t*) throughout. Such a model has to incorporate not only interference from each preceding test, but also a small measure of interference (“self-interference”) from pictures already presented in the current test. This raises two issues that are explored in the “Discussion” section: first, that a particular function for accessibility after time *t* transforms into a different function when “self-interference” is taken into account, and that the functions examined by Rubin and Wenzel (1996) relate to the experimental measure (amount remembered) rather than simply accessibility.

## The experiment

### Participants

Thirty-nine undergraduates at the University of Cambridge (friends of the experimenters)^1^ viewed 1,000 colour slides and, after various intervals of time, were asked to identify pictures they had seen before from a selection, 100 from the original set of 1,000 and 100 New. All participated on a voluntary basis, and the experiment was conducted in accordance with the ethical standards of the 1975 Helsinki declaration. The participants were screened for colour blindness using the Ishihara test. They were each paid £10 on completion of the experiment.^2^

### Stimuli

There were 10 tests in all, requiring a total of 2,000 colour slides. These slides were assigned at random to the inspection series and to be distractors, likewise assigned at random to the different tests. So far as there was a choice, the pictures were “individually memorable and collectively of low similarity and confusability,” following Standing (1973). All participants viewed the same 2,000 pictures for the obvious practical reason.

### Procedure

The experiment began at 6 p.m. on a Friday evening. After some instructions and preliminary training (below), the stimuli were presented to all the participants as a group. The stimuli were projected onto a screen using a carousel projector that stepped on automatically every 6 s. After every 80 slides there was a longer pause (not measured) while the carousel was changed. The first test followed immediately on the presentation of the stimuli. The participants then had supper in the laboratory and were tested twice more that same evening after intervals of 1 and 2 hr. (Taking account of the time needed to first present the stimuli and then the test pictures, the *average* delay before the immediate test equates to 1 hr 3 min.) The participants returned the following morning (Saturday, 10 a.m.) for a further test and thereafter at the intervals listed below:

Immediate (act. 1 hr 3 min average)
1 hr (act. 2 hr 3 min)
2 hr (act. 3 hr 3 min)
16 hr (the following morning)
72 hr (3 days later)
168 hr (1 week later)
264 hr (11 days)
456 hr (19 days)
1,632 hr (beginning of the following term)
2,832 hr (end of the following term)

Except for the test on Saturday morning, all subsequent tests began at 6 p.m. on the day in question.

These test sessions were scheduled (as far as possible) at geometrically increasing intervals, but, of course, had to be at times when the participants (university students) could reasonably make themselves available (in particular, at the beginning and end of the following term). A geometric sequence was approximated because classic studies of forgetting (e.g., Boreas, 1930; Finkenbinder, 1913; Luh, 1922; Strong, 1913 and Ebbinghaus, 1885/1964, as replotted by Woodworth & Schlosberg, 1955, p. 727; cp. Figure 2) show an approximately logarithmic decreasing trend, and equal spacing along that trend line was deemed likely to achieve maximum discrimination between different candidate functions. However, the deviations from perfect geometric spacing proved instrumental in exposing the interference from previous tests.

Each test consisted of 200 slides, of which 100 had been in the original inspection series and 100 were New. Participants were asked to categorise each test slide as

Definitely Old
Probably Old
Possibly Old
Possibly New
Probably New
Definitely New

(adapting the rating procedure introduced by Swets et al., 1961) by ticking one of six boxes on a printed answer sheet. Each test slide was shown for 7 s,^3^ and after each seventh slide there was a blank screen for 7 s to help the participants keep in step with the successive rows on the answer sheet, which also had a blank line after every 7 rows. As a preliminary and before presentation of the stimuli, participants were shown 20 slides of either Oxford or Cambridge and asked to record their confidence, using the six categories above, that it was Oxford rather than Cambridge.

After the experiment was complete, the sequences of stimuli (Old, New) for each test were entered into a computer file, as also were the sequences of confidence ratings (“Definitely Old” . . . “Definitely New”) for each individual participant. Comparison of individual participant’s data with the sequences of stimuli means that the performance of individual participants can be examined. However, anticipating the results a third time, the aggregate data determine the course of forgetting only poorly, and analysis of individual participant’s data would effectively suffer a sixfold increase in standard deviation (*SD*), consequent on the smaller size of the data sample. Indeed, it proved necessary to aggregate the data to discover, first, that each test suffered interference from previous tests (see Figure 2) and, second, that those interference effects could be approximately accommodated by a *t*^−1^ weighting (Table 3). So to have sufficient volumes of data for comparison between the different lapses of time, I have chosen to aggregate all participants’ data prior to analysis.

## Results

Thirty-three of the participants attended every test session and the analysis that follows examines only the data from those 33. For a picture to be recognised as having been seen before, it is necessary for the participant to retrieve an image from memory and match it to that picture. The purpose of the experiment was to discover the relation *f*(*t*) between lapse of time *t* and the accessibility (probability of retrieval) of that image in memory. Analysis is much simplified by splitting it into two parts; first, a model to estimate an accessibility from the categorised data for each lapse of time and, second, a model to relate those estimates of accessibility to the passage of time, taking into account the interference that each test creates for subsequent tests.

### Estimation of accessibility

**Figure 1.**
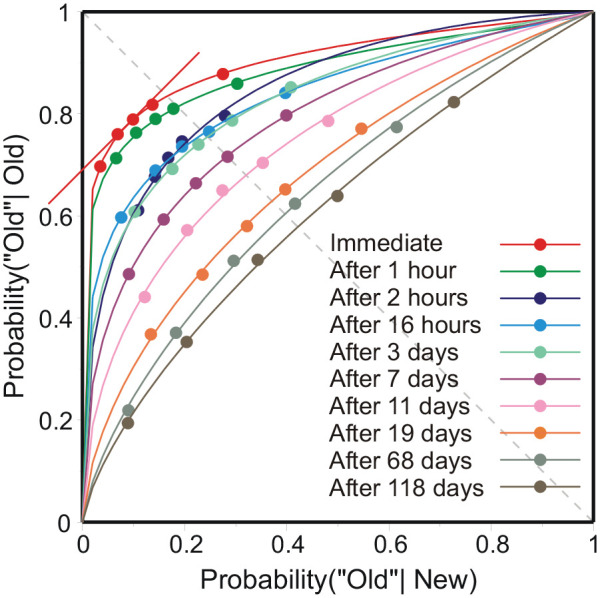
Signal-detection plot for all 10 lapses of time with normal, unequal-variance, operating characteristics fitted to the data. The estimate of accessibility, the maximum value of P(“Old”|Old)–P(“Old”|New), is attained where the tangent (on the “Immediate” characteristic) has gradient 1.

Figure 1 presents the aggregate data in a signal-detection plot, fit with unequal-variance characteristics. The raw data consist of a sequence of assignments by each individual participant to one of the six categories listed above. Comparing this sequence with the sequence of stimuli generates a 2 × 6 matrix (New, Old × 6 response categories) of assignments. The data were aggregated simply by adding the matrices from individual participants. The data points in Figure 1 are the proportions (“Old”|New, “Old”|Old) at the five boundaries between adjacent categories of assignment. I shall use the maximum value of P(“Old”|Old)–P(“Old”|New), on each characteristic, as the estimate of accessibility. This maximum is attained where the characteristic has gradient 1, exemplified by the tangent to the “Immediate” characteristic in Figure 1. This formula, however, needs to be justified. There are four questions to be answered:

1. Why are the model distributions normal?
2. Why are the operating characteristics asymmetric?
3. Why are the operating characteristics curvilinear?
4. Why does that asymmetry decrease with lapse of time?

#### Why are the model distributions normal?

The experimental task is formally category judgement: 200 pictures, 100 New and 100 Old, to be assigned to one of six ordered categories, according as they are deemed to have been seen before. Analysis of sequences of category judgements (cp. also log numerical assignments in magnitude estimation; e.g., Baird et al., 1980) shows, first, that each stimulus is judged relative to its predecessor and, second, that the comparison of one stimulus difference with another is no better than ordinal (Laming, 1984, 1997, pp. 128–130; Stewart et al., 2005). If one picture is judged more likely than its predecessor to have been seen before, it is assigned to a more confident category; if it is judged less likely, it is assigned to a lesser category; and some pictures will be assigned to the same category as the previous picture. So, the category to which a particular picture is assigned is the cumulative sum of small adjustments over preceding trials, and the distribution of judgements over the six categories may be approximated by a normal distribution.

#### Why are the operating characteristics asymmetric?

The operating characteristics in Figure 1 have the same shape as those for detection of an increment in sensory input; for example, the detection of a brief flash of light superimposed on a large uniform luminance (Swets et al., 1961) or detection of a 500-Hz tone added to a background of noise (Watson et al., 1964). Such a flash of light may be visibly detected on “signal” trials, but no similar identification is possible on “noise” trials, when nothing happens. This asymmetry between “signal” and “noise” trials generates the asymmetry in the operating characteristic and points to a similar asymmetry here between Old pictures and New. This explanation is similar to the invocation of a high threshold for recognition by Yonelinas (1994).

#### Why are the operating characteristics curvilinear?

Random selection of test pictures means that, except in the case that an Old picture is recognised as such, there is no difference, between Old and New, in the relation between test picture and the content of memory. In the absence of recognition, both Old and New deliver the same assignments, represented by the distribution (*p_i_*, *i* = 1, . . . 6) in the top row of Table 1 (which illustrates this argument). But if a proportion *a* of Old pictures were recognised and assigned always to “Definitely Old,” the assignment of Old pictures would be as in the second row of Table 1. The operating characteristic would then be linear, because the first two rows are strictly proportional for all columns after the first. The curvilinearity of the operating characteristics implies that not all Old pictures recognised as such are assigned to “Definitely Old.”

**Table 1.** Probabilities of assignment of New and Old pictures to categories, showing how a curvilinear characteristic may be generated.

Test picture	“Definitely Old”	“Probably Old”	“Possibly Old”	“Possibly New”	“Probably New”	“Definitely New”
New pictures	*p*_1_	*p*_2_	*p*_3_	*p*_4_	*p*_5_	*p*_6_
Old pictures	*a* + (1 − *a*)*p*_1_	(1 − *a*)*p*_2_	(1 − *a*)*p*_3_	(1 − *a*)*p*_4_	(1 − *a*)*p*_5_	(1 − *a*)*p*_6_
Old pictures*	*a*_1_ + (1 − *a*_1_)*p*_1_	*a*_2_ + (1 − *a*_1_−*a*_2_)*p*_2_	*a*_3_+(1 − *a*_1_ − *a*_2_−*a*_3_)*p_3_*	(1 − *a*_1_− *a*_2_−*a*_3_)*p*_4_	(1 − *a*_1_− *a*_2_−*a*_3_)*p*_5_	(1 − *a*_1_− *a*_2_−*a*_3_)*p*_6_

Returning to the incremental assignment of pictures to categories (above): if an Old picture is recognised as such, it is certainly not deemed *less* likely than its predecessor to have been seen before and, except in the case that its predecessor was also recognised, will be assigned to a more confident category. Recognition does not guarantee assignment to “Definitely Old,” because assignment is still relative to the preceding judgement, which may have assigned a picture to a low category of confidence. Recognition merely forces an increase in category assignment and migrates recognised pictures towards higher categories of confidence. Suppose that migration directs proportions *a*_1_, *a*_2_, and *a*_3_ to the first three columns in Table 1, so that instead of having a proportion (*a*) of recognitions concentrated in the leftmost column, recognitions are distributed over several adjacent columns. Adding a further row (Old pictures*) to that table shows, on comparison with the top row, that the relation between the probabilities of New and Old is no longer linear.

##### Direction of asymmetry

The columns in Table 1 correspond in order, left to right, to the data points in Figure 1. The assignment *a*_1_, *a*_2_, and *a*_3_ of Old pictures to “Definitely Old,” “Probably Old,” “Possibly Old” in the first three columns increases the ordinates of the leftmost data points by *a*_1_, (*a*_1_ + *a*_2_), and (*a*_1_ + *a*_2_ +*a*_3_) respectively. That upwards displacement of the data points warps the operating characteristic, and that warp is accommodated by an increased variance to the “signal” distribution. Hence the direction of the asymmetry.

##### Estimation of accessibility

Continuing the illustration in Table 1, the difference P(“Old”|Old)–P(“Old”|New) will take the increasing values *a*_1_, (*a*_1_ + *a*_2_) and (*a*_1_ + *a*_2_ +*a*_3_) at those first three data points. Accessibility, the total proportion of Old pictures recognised, can therefore be estimated from the maximum value of P(“Old”|Old)–P(“Old”|New) along each characteristic. That maximum is attained where the characteristic has gradient 1 (the tangent to the “Immediate” characteristic in Figure 1).

**Figure 2.**
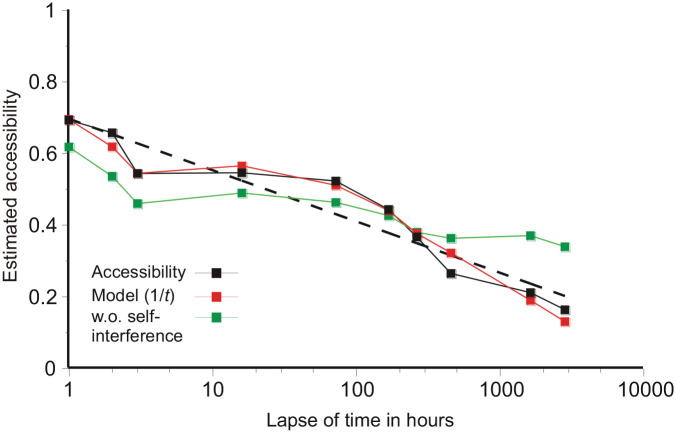
Estimates of accessibility for each lapse of time against a logarithmic abscissa with predictions from the loss model (Equation 2 below), with *f*(*t*) = 1/*t*. The red squares are model predictions based on a reciprocal function and the green squares the same model with self-interference (*s*) set to 0. The black dashed line is the best-fitting logarithmic trend.

Figure 2 plots the estimates of accessibility against a logarithmic abscissa. The straight dashed regression line re log_10_(time) shows that the decreasing trend in accessibility is approximately logarithmic (compare data from Boreas, 1930; Finkenbinder, 1913; Luh, 1922; Strong, 1913 and Ebbinghaus, 1885/1964, as replotted by Woodworth & Schlosberg, 1955, p. 727), but there are large variations about that trend. Inspection shows that the decrement in accessibility from one test to the next is greatest when the second test follows closely (in log time) on its predecessor.

**Figure 3.**
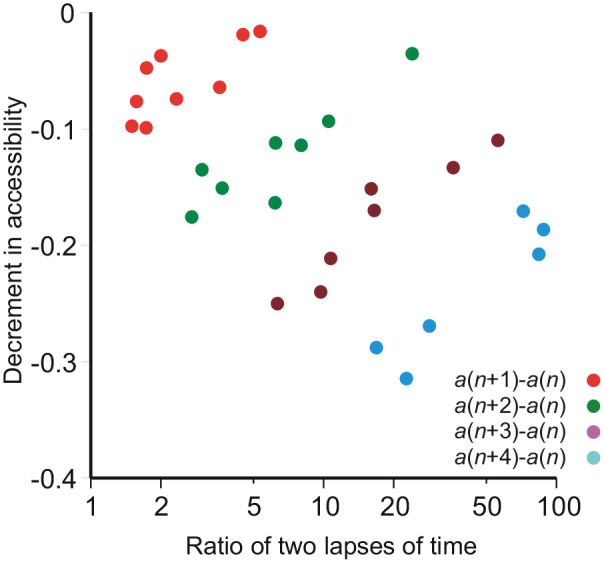
Decrements in accessibility, comparing test (*n* + *k*) with test *n* for *k* = 1, 2, 3, 4, against the ratio of their respective delays.

Figure 3 exhibits this relationship in detail, showing the decreases in accessibility from one test to the next (red symbols) and for sequences of 2, 3, and 4 successive tests. It compares accessibility at test *n* + *k* (*k* = 1, 2, 3, 4) with that at test *n* for successive tests *n*, in relation to the ratio of lapses of time, *t*(*n* + *k*)/*t*(*n*). The Kendall rank correlations between decreases in accessibility and the ratio of lapses of time are set out in Table 2.

**Table 2.** Kendall rank correlation coefficients for the decrements in accessibility in Figure 3.

Decrement in accessibility	*N*	Kendall rank correlation coefficient	Significance (1-tailed)
*a*(*n* + 1) − *a*(*n*)	9	0.667	.006
*a*(*n* + 2) − *a*(*n*)	8	0.714	.007
*a*(*n* + 3) − *a*(*n*)	7	0.905	.002
*a*(*n* + 4) − *a*(*n*)	6	0.600	.045

Each test interferes with recognition on the following test, but that interference decreases with lapse of time. Given that the loss of accessibility decreases (approximately, see Table 3) as 1/*t*, the accessibility of pictures presented on the preceding test (which, by virtue of the experimental design do not contain a match to any of the pictures presented on the present test) decreases faster than that of the original stimulus presentation, so that the effective magnitude of the interference decreases with lapse of time. This accords with Jost’s (1897) law. The interference from preceding tests clearly needs to be taken into account.

**Table 3.** Statistical evaluation of accessibility (Equation 2) with respect to nine candidate loss functions.

Model	Equation	Parameter estimate	*d*/*a*	*s*/*a*	Sum of squares	*r* ^2^	*D.F*.	*F* ratio	Signif.
Sum of squares					0.305		9		
Reciprocal	*f*(*t*) = 1/*t*		4.421E-04	0.002	0.298	0.977	2	150.366	<.001
Residuals					0.007		7		
Inverse square root	*f*(*t*) = 1/√*t*		3.710E-04	0.059	0.292	0.959	2	81.714	<.001
Residuals					0.013		7		
Power	*f*(*t*) = *t*^−α^	0.828	4.322E-04	0.006	0.299	0.982	3	109.323	<.001
Residuals					0.005		6		
Hyperbolic	*f*(*t*) = 1/(c + *t*)	0.650	4.575E-04	0.002	0.299	0.979	3	93.212	<.001
Residuals					0.006		6		
Hyperbolic re √*t*	*f*(*t*) = 1/(c + √*t*)	−0.723	3.599E-04	0.062	0.294	0.966	3	56.117	<.001
Residuals					0.010		6		
Logarithmic	*f*(*t*) = c − ln(*t*)	8.589	7.982E-05	4.144	0.286	0.939	3	30.794	<.001
Residuals					0.019		6		
Logarithmic Sq.	*f*(*t*) = [c − ln(*t*)]^2^	9.651	3.266E-04	14.817	0.294	0.963	3	51.422	<.001
Residuals					0.011		6		
Exp re √*t*	*f*(*t*) = exp(−α√*t*)	0.020	1.140E-03	−0.816	0.265	0.871	3	13.452	.005
Residuals					0.039		6		
Stable model of order ½	*f*(*t*) = (α/√2π).*t*^-3/2^ exp{−½α^2^*/t*}	1.926	5.037E-04	0.000	0.295	0.968	3	60.266	<.001
Residuals					0.010		6		

#### Decrease of asymmetry with lapse of time

**Figure 4.**
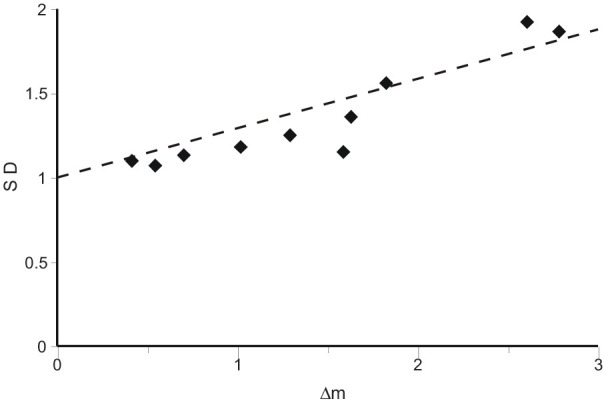
The relation between the excess of the *SD* of the “signal” distribution over 1.0 and the difference of mean (Δm) in the, unequal-variance, operating characteristics fit to the data in Figure 1. The trend line (through 0, 1.0) has gradient 0.275.

The proportion of Old pictures recognised as such simply decreases with lapse of time. Figure 4 shows the relation between difference of mean (Δm) and *SD* in the recognition model relative to a New distribution with mean 0 and *SD* 1. The two are closely related; as the difference of mean increases, so also does the *SD*, indicating increased asymmetry (Kendall Rank correlation (*N* = 10) = 0.822, *p* = .0005). If the *SD* (of the “signal” distribution) were 1.0, the model would be symmetric. The excess over 1.0 increases as 0.275 of the increase in mean (Δm) (Green & Swets, 1966, p. 98, report a value of 0.25, approx. See also Ratcliff et al., 1992).

The sequence of data points in Figure 4 corresponds (right to left) to the sequence of tests (only “After 2 hours” is out of sequence), so that asymmetry decreases progressively as recognition gets weaker. Such a relationship with accuracy has previously been reported by Glanzer et al. (1999) and Wais et al. (2006).

### Loss of accessibility with time

**Figure 5.**
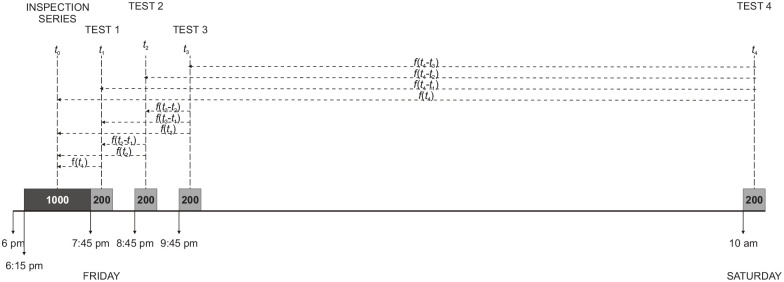
A linear timeline for the first four tests. “1,000” (6:15 to 7:45 p.m.) is the original presentation of the inspection series; *f*(*t*) describes the progressive loss of accessibility. “200” (7:45 onwards) is the first test series; *f*(*t* − *t*_1_) describes its decreasing potential to interfere. Likewise “200” at 8:45 p.m., at 9:45 p.m., and at 10 a.m. on Saturday.

There were 10 tests at times *t_i_*, (*i* = 1, . . . 10). With respect to an origin (*t* = 0) taken to be the middle of the original presentation (about 6:50 p.m. on the Friday evening), the accessibility of those pictures is *f*(*t_n_*) at the *n*th test and the accessibilities of the preceding tests at that same point in time are *f*(*t_n_*− *t_i_*), (*i* = 1, . . . *n* − 1). Figure 5 presents a linear time-line for the first four tests. Presentation of the 1,000 pictures to be subsequently tested by recognition (the inspection series) occupied the period from 6:15 to 7:45. The 200 pictures in Test 1 (beginning about 7:45 p.m.) include 100 from the inspection series and 100 New pictures. At this point in time the inspection series has accessibility *f*(*t*_1_), measured from the mid-point of the original presentation to the mid-point of the test. In Test 2 that comprised another 100 from the inspection series and another 100 New pictures (“200” at 8:45), the accessibility of the inspection series has decreased to *f*(*t*_2_), but pictures viewed or retrieved during the first test increase the population of New pictures that might be retrieved. Those additional New pictures have accessibility *f*(*t*_2_ − *t*_1_), again measured from mid-point to mid-point. In Test 4 the accessibility of the inspection series has decreased to *f*(*t*_4_) and there are three previous tests to generate interference. They have accessibilities respectively *f*(*t_4_*−*t_i_*), *i* = 1, 2, 3.

Suppose now an Old picture to be presented at the *n*th test: Participants have 7 s to view the test picture, and if the original image is retrieved within that time, the test picture is identified as “Old.” Write the derated probability of that retrieval—derated on account of lapse of time—as *f*(*t_n_*), so that on initial presentation a picture has accessibility 1. If that original image is not retrieved, the participant may instead retrieve one of the other 999 original images—write that probability as 999*df*(*t_n_*) (*d* for “distractor”)—or one of the 200 images from test *i, i* = 1, *n* − 1, with probability 200*df*(*t_n_* − *t_i_*), or even one of the test pictures previously presented on the *n*th test (which took about 30 min) with probability *s* (self-interference). Assuming that one of these retrievals occurs—there were only a few failures to respond at all—the conditional probability that the original image is retrieved is

(2) $$p_{n}\;=\;f(t_{n})/[(1+999d)f(t_{n})+200d\sum_{1}^{n-1}f(t_{n}-\;t_{i})+s].$$

This (*p_n_*) is the quantity that is matched to the estimated accessibilities. The red symbols in Figure 2 show the model predictions with *f*(*t*) = 1/*t*. Self-interference from the present test (*s*) is an essential term in this equation. Suppose, instead, that *s* = 0; then, in the absence of intervening tests, *p_n_* = 1/(1 + 999*d*), and there is no forgetting, because *f*(*t_n_*) cancels. The green symbols in Figure 2 show predictions from the same model with self-interference set to 0.

Table 3 presents a statistical evaluation of this model for loss of accessibility with respect to nine candidate *f*(*t*)’s. These include the four functions that Rubin and Wenzel (1996) found to fit the best (see “Introduction”) and five similar. The candidate functions are listed here with only one free parameter, whereas Rubin & Wenzel’s functions had two. The difference is illusory, because Equation 2 introduces an additional parameter (*s*, self-interference). The retrieval of the unique target image at any time during the 7 s allowed for responding is represented by the model function *f*(*t*). The retrieval of some other distracter image is *df*(*t*). In comparison, *d* is tiny, about 5 × 10^−4^, but needs to be multiplied by the number of other images that might be retrieved (upwards of 999). The entry “0.305” is the sum of squares of the individual accessibilities in Figure 2 relative to their mean. The evaluation of the nine candidate functions turns on what further reduction each can effect in that sum. This further reduction is calculated by minimising the sum of squares, using Excel’s Solver routine. The significance levels in the rightmost column means that each loss function accounts for a highly significant proportion of the variation of accessibility with lapse of time (of course); *r*^2^ for the different models varies from 0.871 to 0.982.

Seven of the loss functions involve different formulae, each with its own free parameter, and there is no valid statistical comparison between them. But two of the functions (reciprocal and inverse square root) are the power and hyperbolic functions (or hyperbolic wrt √time) with particular parameter values inserted. In no case does the additional parameter offer a significant improvement in fit.

## Comparisons with previous studies

Experiments that track the recognition of unique images (pictures) exhibit a different relation with respect to time to that revealed by analyses of aggregate performance (recognition, recall of nonsense syllables, words, poetry, etc.) in complete experiments. Depending on the method of analysis, the present experiment exemplifies both relations.

### Recognition of unique images

**Figure 6.**
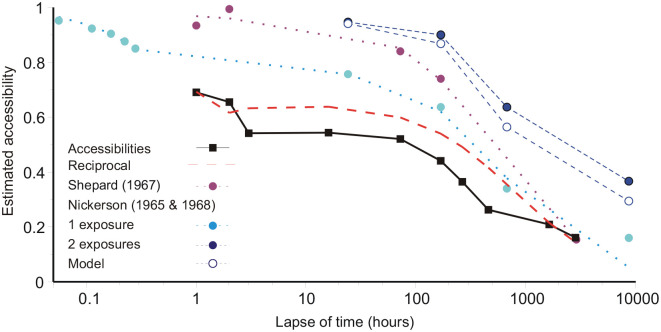
Accessibilities from Shepard (1967) and Nickerson (1968) compared with the present experiment. The functions fit to these data are Equation 4 with *f*(*t*) = 1/*t*, which adjusts for interference from the initial test. The data were obtained from Table 1 in Shepard (1967) and by using the Acrobat measurement tool on bitmaps of Figure 1 in Nickerson (1965) and Figure 1 in Nickerson (1968).

The present experiment was originally designed as an improvement on the study by Shepard (1967), with particular attention to recognition after a 2-hr delay. The size of the inspection series was increased from 612 to 1,000, and the test series from 68 2AFC pairs to 200 single pictures combined with a 6-point rating scale. After an immediate test, Shepard retested *different* groups of four participants after intervals of up to 120 days, with the results presented in Figure 6. Shepard’s data are presented here as accessibilities, *not* proportions correct, where, in a test with equal numbers of New and Old pictures,

(3) Accessibility=2×Proportioncorrect−1,

so that an accessibility of 0 (the participants have not seen any of the pictures before) equates to 50% correct (the participants are guessing). The apparent improvement in recognition after 2 hr was not replicated; instead, performance was depressed by interference from the previous “Immediate” test.

Figure 6 also shows data from Nickerson (1965, 1968). Nickerson (1965) asked 56 participants to look through a series of 600 black-and-white photographs at one per 5 s. The first 200 photographs were all different, but thereafter some photographs were repeated at intervals (lags) of 40, 80, 120, 160, and 200 photographs. Figure 6 shows estimated accessibilities for delays of 0.056 to 0.279 hr. Nickerson (1968) then retested *different* sub-groups of those participants after four different intervals, a day, a week, a month, and a year. Figure 6 shows separate accessibilities for pictures presented once by Nickerson (1965) and those presented twice. Accessibilities for twice-presented pictures pose a problem.

By analogy with single images, a picture twice-presented should be recognised as “Old” if either of the two identical images is retrieved. If a single image has accessibility *a*, then, assuming the retrievals to be independent, the accessibility of a twice-presented picture should be 1−(1 − *a*)^2^. These predictions, for twice-presented pictures, are shown in Figure 6 labelled as “Model.” In fact, accessibilities for twice-presented pictures are systematically greater than this; this result and the method of testing are set out in the online Supplementary Material. The predictions for twice-presented pictures can be increased only if the retrievals of the two images are *negatively* correlated.

Comparing Shepard’s and Nickerson’s data with the present experiment, the chief difference is that the 2,000 pictures here were less distinguishable. Of necessity, 2,000 colour slides come from a much smaller number of sources and slides from the same source tend to have relative similarities. Looking at the immediate tests, mean accessibility was 0.69, compared with 0.934 (Shepard, 1967) and 0.85–0.95 (Nickerson, 1965). The number of pictures matters, but not that much. Standing (1973) reported 83% correct (mean accessibility 0.66) after learning 10,000 pictures.

Shepard (1967) and Nickerson (1965) both tested different groups of participants after each delay. In each experiment there was an immediate test, which would have interfered with subsequent recognition, but no further interference was generated thereafter. Modifying Equation 1, the equivalent formula for the present experiment would be adjusting for the interference from the immediate test at *t*_1_ only and self-interference from the current test. Substituting *f*(*t*) = 1/*t* and the parameter values from Table 3 give the characteristic labelled “reciprocal” in Figure 6 (interference from tests after the first being ignored). But the same equation applied to the accessibility data from Shepard (1967) and Nickerson (1965, 1968), with the numerical constants in Equation 4 appropriately substituted (see Table 4), fits well. The parameter values for the Shepard and Nickerson data were estimated by least squares, and it is worth noting that self-interference, relative to the accessibility of an original image, is the same in all three analyses.

(4) $$p_{n}\;=\;f(t_{n})/[(1+999d)f(t_{n})+200df(t_{n}-\;t_{1})+s],$$

**Table 4.** Comparison between the present experiment, Shepard (1967) and Nickerson (1965, 1968).

Source	Number of pictures in stimulus set	Number of pictures in test	*d*	*s*
Present experiment	1,000	200	4.42 × 10^−4^	1.91×10^−3^
Shepard (1967)	612	68	5.10 × 10^−5^	1.93×10^−3^
Nickerson (1965, 1968)	400^a^	200	4.41 × 10^−4^	2.06×10^−3^

a Number of distinct images.

### Aggregate performance in complete experiments

**Figure 7.**
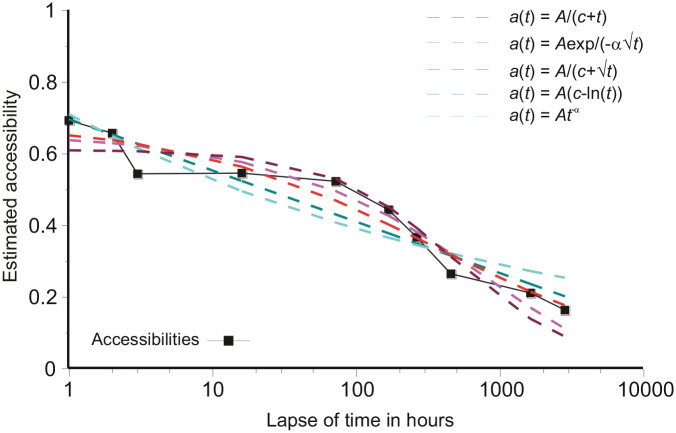
Five candidate functions fit to the present data ignoring interference.

Rubin and Wenzel (1996) fit 105 different two-parameter functions to each of 210 published sets of data. They used “standard curve-fitting techniques” (p. 737), which I take to mean estimation of model parameters by least squares. Using historic data, there was no opportunity to make any adjustment for the detailed design of the source experiments. Figure 7 shows the same procedure applied to the present data, that is, with no adjustment for interference from previous tests, for Rubin and Wenzel’s five best-fitting functions (the logarithmic square duplicates the logarithmic model). The parameter estimates for this unadjusted fit are shown in Table 5. The logarithmic function duplicates the trend line in Figure 2 and, by choosing optimal parameter values, a variety of quite different functional forms can be made to approximate that trend line over the temporal range of this experiment. That dependence on parameters is highlighted by the values for the hyperbolic parameters in Table 5. The psychological significance of these functional forms is doubtful, but possibly the logarithm reflects the custom in such experiments to space the different tests equally in a logarithmic metric.

**Table 5.** Statistical evaluation of accessibilities ignoring interference.

Model	Equation	*A*	Parameter estimate	Sum of squares	*r* ^2^	*DF*.	*F* ratio	Significance
Sum of squares				0.305		9		
Hyperbolic re *t*	*f*(*t*) = 1/(c + *t*)	288.133	473.677	0.276	0.905	2	33.219	<.001
Residuals				0.029		7		
Exp re √*t*	*f*(*t*) = *A*exp(−α√*t*)	0.658	−0.034	0.285	0.935	2	50.344	<.001
Residuals				0.020		7		
Hyperbolic re √*t*	*f*(*t*) = *A*/(c + √*t*)	12.465	18.208	0.288	0.944	2	59.402	<.001
Residuals				0.017		7		
Logarithmic	*f*(*t*) = *A*[c − ln(*t*)]	0.062	0.694	0.279	0.916	2	38.379	<.001
Residuals				0.025		7		
Logarithmic Sq.	*f*(*t*) = *A*[c − ln(*t*)]^2^	−0.062	0.700	0.279	0.916	2	38.379	<.001
Residuals				0.025		7		
Power	*f*(*t*) = *At*^-α^	0.708	−0.130	0.262	0.859	2	21.340	.001
Residuals				0.043		7		

Comparing Figures 6 and 7, if the data track recognition of a unique image in memory (Nickerson, 1965, 1968; Shepard, 1967) Equation 4, with *f*(*t*) a reciprocal or similar, fits well; and a reciprocal also fits the present data well if full account is taken of the interference from successive tests (Figure 2). The functions singled out by Rubin and Wenzel are inferior (Table 3). If, instead, merely the amount recalled is analysed, with no adjustment for interference within the experiment and possibly multiple sources for each recall, then the functions singled out by Rubin and Wenzel are best and the functions that work well with unique images (e.g., the reciprocal in Figure 6) are nowhere. In short, Figures 6 and 7 exhibit two different relations. The difference could, of course, be a matter of pictures versus other kinds of material. However, the present study can be fit into both comparisons, depending on the method of analysis, showing that this distinction is a matter of the question asked of the data.

If model predictions using the reciprocal are substituted for the actual accessibilities in Figure 7, the analytical results with respect to Rubin and Wenzel’s (1996) functions are almost unchanged. It follows that, for this present experiment, the difference (between Figures 2 and 7) is entirely consequent on ignoring the interference from previous tests. In general, the pattern of interference in an experiment will depend on its design, and this is arguably the reason why Rubin and Wenzel obtained so wide a variety of analytical results. Tracking the retrieval of a unique image (Figure 6) would seem to give the clearer insight into forgetting.

## Discussion

Thirty-three participants viewed 1,000 pictures for 6 s each. Recognition was tested after 10 different intervals of time by mixing 100 of the original 1,000 with 100 new pictures. Participants judged “Old” or “New” on a 6-point scale. Analysis of their aggregate data required two models in cascade: first, a model to estimate accessibility after each lapse of time, the normal unequal-variance model, and, second, a model to relate those different estimates of accessibility to the lapse of time, taking into account the interference from intervening tests.

AccessibilityEach set of data points in Figure 1 was fit with a normal unequal-variance recognition characteristic. But the parameters of this model (Δ*m*, difference of means; *s*, ratio of *SD*s) do not relate to any other concepts in memory, and some reinterpretation is needed. Considering (above) how recognition judgements come to be dispersed over the different categories, the model distributions are interpreted as approximate representations of the actual dispersions of “Old” and “New” category judgements and accessibility is estimated from the maximum difference, P(“Old”|Old)–P(“Old”|New), attained on the operating characteristic.

Random assignment of pictures within the experiment means that there is no evidential basis for distinguishing “Old” from “New” except in the case that a picture is recognised as having been seen before. Recognition requires the participant to retrieve a matching image from memory. That probability of retrieval (accessibility) is estimated by P(“Old”|Old)–P(“Old”|New); it is different for different data points, which are analogous to different biases (cp. Swets et al., 1961). But it happens that the maximum difference, P(“Old”|Old)–P(“Old”|New), occurs at the locus where the tangent to the operating characteristic has gradient 1, and the bias between “Old” and “New” is even. That maximum difference is a natural estimator of the probability of recognising an Old picture as “Old.”

But all this impinges on an ongoing controversy between the unequal-variance recognition model and a dual-process model, which says that if a picture is recognised as “Old,” it is assigned to “Definitely Old”; in the absence of recognition it is assigned on the basis of some other cue, represented by an *equal-variance* recognition model.

### The unequal variance/dual-process controversy

Wixted (2007) has published a comprehensive review of these two alternatives in relation to a very large body of research. He commented:

Specifically, how can two well-established theories of recognition memory—dual-process theory and signal detection theory—be reconciled? (Wixted, 2007, p. 153)

In truth, there is no problem of reconciliation; the empirical reality is the data points and the operating characteristics that may be fit to them. If recognition of a picture as “Old” could be observed absolutely, independent of its assignment to “Definitely Old”—that would be a different matter. But as things stand, these two models are simply alternative formulations of the same data:

The competition between the UVSD model and the DPSD model is an interesting one because they are both characterized by two parameters, and they both fit ROC data well. (Wixted, 2007, p. 155)

Such conflict as there is centres on how different researchers *talk* about recognition, but that is not an empirical matter. For example, it is near-universally assumed that the distributions in a signal-detection model represent variability intrinsic to the stimuli. When that model is applied to recognition memory, there has to be some corresponding source of variability—“memory strength.” “Memory strength” is greater for Old stimuli, because recognition exceeds chance, and is also more variable, because the operating characteristic is asymmetric. In the dual-process model some Old stimuli are recognised absolutely (if “recollection” exceeds a threshold); otherwise identification depends on “familiarity,” which is the variable in the equal-variance signal-detection component (Yonelinas, 1994). A signal-detection model has to be incorporated, because the operating characteristic is curved, and “familiarity” is the additional cue needed to assign some Old stimuli to categories less extreme than “Definitely Old.” Discussions of “Memory strength,” “recollection,” and “familiarity” treat them as empirical constructs, but, in truth, they are no more than different ways of talking about recognition. It is a matter of whether one cracks one’s egg open at the big or the little end.

Sets of five related operating points, as in Figure 1, are quite insufficient to identify the underlying process, and the choice of model is to some extent arbitrary. The normal, unequal-variance model is the most common choice because it is so widely known. But Blackwell’s (1953) high-threshold model has been resurrected (Kellen et al., 2013) and Snodgrass and Corwin (1988) have proposed a double threshold model with the second threshold identifying a New stimulus absolutely. A more intelligent choice would be the ideal observer model for the signal of a sample of noise (Green, 1960; Green & Swets, 1966 pp. 174–175). This has a continuous operating characteristic that is naturally asymmetric. Depending on the model, the estimate of accessibility, the maximum of [P(“Old”|Old)–P(“Old”|New)], is slightly different. But this matters little in the analysis of forgetting below; preliminary calculations showed that even the average of [P(“Old”|Old)–P(“Old”|New)], averaged across each set of five data points, worked well.

The argument (above) leading to the estimate of accessibility turns the modelling inside out. It begins with a process (category judgement) that disperses the judgements among the different categories. The model distributions are then simply approximate representations of that distribution of judgements. The asymmetry of the data tells us that some Old pictures are recognised absolutely. But the curvature of the operating characteristics tells us, also, that not all such recognitions are assigned to “Definitely Old.” The key to all this is the relativity of category judgements—each judgement being based on its predecessor as a point of comparison (Laming, 1984, 1997, pp. 128–130; Stewart et al., 2005). Relativity by itself would deliver a symmetric, curvilinear, operating characteristic, but the recognition of a picture as Old (all pictures are New on first presentation) skews the subsequent category assignments.

### Loss functions

The estimates of accessibility have been fit with nine different loss functions (see Table 3), those preferred by Rubin and Wenzel (1996) and several similar. The loss functions are entered into an equation (Equation 1) that adjusts for the interference from intervening tests and also self-interference from the current test. All nine functions capture the greater part of the loss of accessibility with lapse of time (*r*^2^ ranges from 0.871 to 0.982). The best-fitting functions in Table 3 are the power law *f*(*t*) = *t*^−α^, with α = .828; the hyperbola *f*(*t*) = 1/(*c* + *t*), with *c* = 0.650 (hr), and the reciprocal, but the experimental data admit, as it were, two degrees of freedom. First, the power law exponent is uncertain (results for *f*(*t*)=1/√*t* and *f*(*t*)=(α/√2π).*t*^-3/2^exp{−½α^2^*/t*} suggest an exponent in the range (−1.5, −0.5)) and, second, the offset from the origin of the timescale (the joint success of the hyperbolic and the reciprocal) show that the additive constant *c* is also poorly determined. Seven of the functions involve a free parameter, with no valid statistical comparison between them. But two of them (reciprocal and inverse square root) are special cases of the power model, setting the power exponent to –1 and –0.5, respectively. The additional reductions in the residual sum of squares achieved with an optimal choice of exponent are not significant.

The question arises whether the lack of discrimination between different loss functions is consequent on the need to model the interference. Table 5 shows the results of fitting Rubin and Wenzel’s preferred functions in Figure 7 without any adjustment for interference. Again, discrimination between the different candidate functions is poor. This happens because the insertion of optimal parameter values can make a variety of different functional forms approximate the data over the experimental range and a lack of discrimination results. Modelling the interference makes little difference.

Rubin and Wenzel’s functions all have two free parameters, while the functions listed in Table 3 have only one. The difference is illusory, because the functions listed here are entered into a formula for probability of retrieval (Equation 1) that involves one additional free parameter (self-interference). The notion of “self-interference” ought not to surprise. If the pictures presented on a previous test can cause interference, then so too can those previously presented during the present test. Such interference is demonstrable in free recall (Laming, 2006, 2008)

Recently, Fisher and Radvansky (2018) have published an experiment on the forgetting of words and narrative material over six different delays up to 12 weeks. The words were tested by recall (though intrusions were not reported), the narrative material by Yes/No recognition of a probe sentence (false-positives again not reported. In the absence of false-positives, accessibility cannot be estimated.). Fisher and Radvansky suggest that there is a shift in the pattern of forgetting after about 7 days, but in their experiment each different delay was based on data from a different sub-group of 48 participants and the general character of their loss functions is logarithmic (cf. Figure 2 and Woodworth & Schlosberg, 1955, p. 727).

#### Jost’s law

As stated by Woodworth (1938, pp. 58–59),

If two associations are now of equal strength but of different ages, the older one will lose strength more slowly with the further passage of time.

and (a second law)

If two associations are of equal strength and different ages, further study has greater value for the older one.

These are simply qualitative generalisations. It is not clear whether the second law prescribes a specific enhancement of the effects of “further study” on an existing association or whether it merely reflects a natural advantage, consequent on its slower loss of strength, when that association is next tested.

If accessibility decreases as 1/*t* (or 1/√*t* or ln *t*), the relative strength of two associations of ages *t*_1_ and *t*_2_ decreases as (*t*_1_/*t*_2_)^−2^ or ((*t*_1_/*t*_2_)^−1.5^ or (*t*_1_/*t*_2_)^−1^ and all of the functions explored above conform to Jost’s generalisation. But if accessibility decreases as *e*^−*bt*^, the relative strength decreases as exp{−*b*(*t*_1_ – *t*_2_)}, which is constant as *t*_1_ and *t*_2_ increase with *t*_1_—*t*_2_ constant, independent of the ratio *t*_1_/*t*_2_. Moreover, if the functional decrease *were* exponential, then depending on the exponential parameter, the volume of material accessible from memory would reach an asymptote. Results from the Brown–Peterson paradigm (e.g., Laming, 1992), for example, suggest that such an asymptote would be reached very quickly, in a matter of minutes.

Examination of this issue (e.g., Wixted, 2004) is confounded with extra-experimental assumptions. For example, that memories “fade” or “break up” or become progressively less distinct (Woodworth, 1938, p. 51), so that recall fails because the memory is no longer there to be recalled; or that there is an internal association between the cue and the desired response of decreasing strength. Or memories fail to be recalled because of obstruction by other memories (McGeogh, 1932), and that this process is mediated by the cue to recall acquiring additional internal associations (“cue-overload,” Watkins & Watkins, 1975). Or even that recall is dependent on the agency of a cue.

When participants fail to recall the desired stimulus, they commonly produce other responses instead. So there are multiple memories available for recall and there must be some process to select between them. Looking at experiments over periods of minutes, that process shows a strong bias towards the most recent events. The loss functions that have been evaluated here over a long delay could all serve as representatives of that process in the short term and there is, as yet, no empirical reason why the same process should not encompass an entire lifetime’s record of memories. But the variety of mathematical functions that work, more or less, means that present experimental methods are too imprecise—whence the recourse to extra-experimental assumptions.

#### The reciprocal relation

The particular virtue of the reciprocal is that it fits both the present experiment and Shepard (1967) and Nickerson (1965, 1968) well and, at the same time, is an especially simple vehicle for calculation in the context of more elaborate models. But it should not be supposed that the reciprocal is definitive; it merely characterises the general course of forgetting.

This experiment was conceived in the belief that more detailed knowledge of the relationship between lapse of time and accessibility would be informative with respect to memory function. Nine different functions have been examined and they all fit well. That analysis is based on a large volume of data (66,000 observations) and it is unlikely that a greater number of observations or testing after additional delays (within the present range) would discriminate further. Each function has two or three free parameters. When optimal values are assigned to those parameters (different values in different functions), they generate numerical predictions very close to each other and equally close to the data (seven functions have *r*^2^ values in excess of 0.95). Additional observations and test delays will not change those numerical predictions. The lack of discrimination results simply from inserting optimal parameter values in quite different functional forms. Nevertheless, the reciprocal presents a problem. It has to be presumed that only one picture can be retrieved at a time, so that the integral of accessibility from 0 to infinity with respect to time, after suitable scaling, must be 1. One can define a probability of retrieval after some time *t* and conduct an experiment to estimate that probability. One can repeat that exercise for different lapses of time. But the idea of a probability distribution over all preceding events recorded in memory is not tenable because *t*^−1^ is not integrable from 0 to infinity—as are other functions in Table 3—and so cannot be scaled to a probability distribution.

Suppose instead each sensory input to be added to a pre-existing substrate. A test picture is recognised as “Old” on the basis of its correlation with that substrate, as at the time of test. Represent the picture by a random variable *X* of great dimensionality, and its combination with the substrate pre-existing at time of presentation (which might influence the perception of the picture), by *X*_0_. Obviously, the test picture *X* will correlate more than sufficiently with *X*_0_. In between presentation and test there will be many further sensory inputs, *x_i_*, *i* = 1, 2 . . . and so on, added to the combination of substrate and picture, so that at time of test the substrate has evolved to $X_{0}+\overset{n}{\underset{1}{\Sigma }}x_{i}$ for some large number *n*. Covariance with the original picture presented a second time is $\left[1+\overset{n}{\underset{1}{\Sigma }}E\{x_{i}X\}\right]$ , and the probability of recognition is of the form [1 + *n*ρ]^−1^ for some small constant ρ (cp. Metcalf & Murdock, 1981, Eq. 7, p. 165). Since the sensory inputs *x_i_* are a sequence presented in course of time, that probability transposes into (1 + ρ*t*)^−1^, which is the hyperbolic model in Table 3.

#### The stable density of order ½

The function *f*(*t*) = (α/√2π).*t*^−3/2^exp{−½α^2^*/t*} was included precisely because it was a probability density. “Stability” means that the sum of *n* independent variates $\overset{n}{\underset{1}{\Sigma }}X_{i}$ can be scaled so that it has the same distribution as its parent. In the present case the scaling factor is *n*^-2^ (Feller, 1966, p. 51); that is, $n^{-2}\overset{n}{\underset{1}{\Sigma }}X_{i}$ is distributed as each *X_i_*. (The normal distribution is also stable, with scaling factor *n*^-½^). The strictly positive stable density of order ½ is also the distribution of *X*^−2^, where *X* is normal (0, α^2^), and has cumulative distribution function $\frac{2}{\sqrt{2\pi }}\int_{\alpha /\sqrt{x}}^{\infty }e^{-½y^{2}}dy$ .

I do not suggest the strictly positive stable density of order ½ as a model itself. The density tends to 0 at *x* = 0 and has a peak at α^2^/3; this would conflict with recall in the short term. But it is the limiting distribution of a number of other processes (first passage times and returns to the origin in random walks—e.g., tossing a fair coin until the number of heads equals the number of tails—and waiting and recurrence times more generally; Feller, 1957, pp. 87, 231). So, some subordinate process with this distribution as its limit might provide a viable model.

### Summary

This experiment was conceived in the hope that knowledge of the functional form of the relation between lapse of time and loss of recall over the medium to long term would be informative with respect to the mechanism of forgetting.

There are two relationships needing to be distinguished. If sufficient care is taken to accommodate the effects of interference within the experiment, the loss function for a unique image (a picture) is approximately reciprocal. If, instead, the amount recalled or recognised is regressed on lapse of time, with no allowance for interference within the experiment and possibly multiple sources for each recall, the relation is approximately logarithmic. The present experiment can be analysed in both ways and, depending on the method of analysis, exhibits the one relation or the other.

The relationship between lapse of time and probability of access of a unique image is poorly determined; it admits, as it were, two degrees of freedom. The data can be accommodated with a power law with a range of different exponents (say −1.5, −0.5) and, at the same time, with a range of different offsets from the origin of the time scale. This possibly happens because the substitution of optimal values for function parameters enables a variety of different functional forms to approximate the data.

Over the long term a reciprocal integrates to infinity with respect to time. It cannot be scaled to deliver a probability distribution, and memory cannot then be represented as a distribution over all previous entries. But a hyperbolic relation *can* be generated by correlation between a test picture and the accumulated content of memory. However, the fit of the strictly positive stable distribution of order ½ shows that such a conclusion would be premature.

## Supplemental Material

sj-docx-1-qjp-10.1177_17470218211047862 – Supplemental material for Forgetting tracked by recognition of pictures Click here for additional data file. Supplemental material, sj-docx-1-qjp-10.1177_17470218211047862 for Forgetting tracked by recognition of pictures by Donald Laming in Quarterly Journal of Experimental Psychology

sj-xls-2-qjp-10.1177_17470218211047862 – Supplemental material for Forgetting tracked by recognition of pictures Click here for additional data file. Supplemental material, sj-xls-2-qjp-10.1177_17470218211047862 for Forgetting tracked by recognition of pictures by Donald Laming in Quarterly Journal of Experimental Psychology

## Notes

1. I thank Conta Rowan Hamilton and Jane Law, who carried out the experiment together as a part of their third-year coursework at the University of Cambridge, Department of Experimental Psychology, in 1983–1984.

2. This was in 1984.

3. The inspection series was presented at one slide per 6 s, but the test series at one slide per 7 s.
