# Supplementary material for: Forgetting tracked by recognition of pictures
Source: Q J Exp Psychol (Hove). 2021 Oct 4;75(4):680–94. doi: 10.1177/17470218211047862 (PMC8915223; doi:10.1177/17470218211047862)
Supplement: sj-docx-1-qjp-10.1177_17470218211047862 – Supplemental material for Forgetting tracked by recognition of pictures [file sj-docx-1-qjp-10.1177_17470218211047862.docx]

Supplementary Material for:

##### **FORGETTING tracked BY RECOGNITION OF PICTURES**

**Donald Laming**

University of Cambridge, Department of Psychology

# Analysis of Nickerson’s (1968) data

Nickerson (1968) tested different groups of 14 participants after four different intervals, a day, a week, a month and a year, on the recognition of previously viewed pictures. Of 200 test pictures, 100 were new, and 100 had been shown to the participants in a previous study (Nickerson, 1965). Of that 100, 50 had been presented once, and 50 twice. The data from each group of 14 participants therefore has the format:

| Stimuli | ‘Old’ | ‘New’ | Total |
| --- | --- | --- | --- |
| New pictures | *x_11_* | *x_12_* | 1400 |
| 1 previous exposure | *x_21_* | *x_22_* | 700 |
| 2 previous exposures | *x_31_* | *x_32_* | 700 |

where *x_ij_* are numerical entries estimated from Figure 1 in Nickerson (1968).

Let P(‘Old’| New picture) = *y*, and the accessibility of a picture after a single exposure be *a*. Then P(‘Old’| 1 previous exposure) = *y*+ *a*; (Eq. 1 above) If the joint accessibility after 2 exposures is [1-(1-*a*)^2^] – this is the hypothesis to be tested – then P(‘Old’| 2 previous exposures) = *y*+ *a*(2-*a*); (Eq. 1 again). The log likelihood ratio in favour of this hypothesis is

ln λ = *x_11_*ln(1400*y/x_11_*)+ *x_12_*ln(1400(1-*y*)*/x_12_*)+ *x_21_*ln(700(*y*+ *a*)*/x_21_*)+

*x_22_*ln(700(1–*y-a*)*/x_22_*)+ *x_31_*ln(700(*y*+ *a*(2-*a*))*/x_31_*)+ *x_32_*ln(700(1–*y-a*(2-*a*))*/x_32_*)

Maximising ln λ, where –2 ln λ is distributed as χ^2^ with 1 d.f., gives these results:

| Time delay | –2 ln λ_max_ | Significance |
| --- | --- | --- |
| 1 day | 0.410 | 0.522 |
| 1 week | 3.979 | 0.046 |
| 1 month | 5.8855 | 0.015 |
| 1 year | 4.090 | 0.043 |
| Sum | 14.364 | 0.006 |
